# Use of Chloroform in Midwifery

**Published:** 1853-05

**Authors:** George N. Burwell

**Affiliations:** Buffalo


*Use of Chloroform in Midwifery.* By George N. Burwell, M. D., of Buffalo.—Total number of cases in which chloroform was given for a greater or less length of time,..................................180

Number of cases where the relief obtained from the chloroform was decided,...........122

Number of cases where the relief was moderate,......................................55

Number of cases where there was no relief,...3

Longest time in which it has been continuously administered to the patient,............14¾ hours.

Average duration of administration to each patient, about ..............................1 "

Number of cases which have been terminated by the forceps,................................17

| | | | |
|---|---|---|---|
| do | do | of craniotomy,............ | 1 |
| do | do | turning, .................. | 1 |
| do | do | first labor,.............. | 88 |
| do | do | still-born child'n (in 13 labors,) .................. | 14 |
| do | do | flooding,................. | 7 |
| do | do | inflammation, recovered,.. | 5 |
| do | do | do died,........ | 1 |
| do | do | bad health, succeeding the confinement,......... | 25 |

Some remarks upon each of these points will be necessary to their full appreciation.

*Of the cases in which the relief was decided.*—Under this head I place those cases in which the pain was always greatly diminished, and occasionally all taken away. In thirty-seven of these cases the patients were entirely insensible to the birth of the children. The remainder not only expressed themselves greatly relieved, but in most instances gave other evidence of it, as by calling for more of the remedy; going to sleep in the intervals of pain, and in many ways appearing quieter and easier. There were frequently in these cases terms of transient insensibility, or of delirum, after a fresh breathing of the chloroform; but I do not for that reason put them in the list of cases of total insensibility, as I place none there to whose insensibility there was not some depth and duration.

*Of the cases in which the relief was moderate.*—Under this head I classify, 1st: A few cases in which there was no call for the remedy; but little diminution of the cries or of the other appearances of suffering, and of course entire sensibility to the birth of the children. These, when taking it, if pressed for an answer, would say it relieved them: if asked if they wanted more, would say yes; but if I discontinued it, would not inquire for it. Were it not that I judged from their actions (they even occasionally going to sleep in the intervals of pain), I should say they obtained no relief. 2nd. I place here some who, on the contrary, have expressed greater relief than I have given them credit for. Judging rather from my impressions from the observation of the cases, and the small quantity of the chloroform they had taken, I concluded they expressed themselves too strongly, and therefore placed them on this list. 3rd. A large proportion of the cases classified here, are those who took the remedy only from five to fifteen or twenty minutes before the conclusion of the labor; and although relieved, had no opportunity of experiencing that decided relief, which the longer use of the remedy would have afforded.

*Of the cases which derived no relief.*—One was a large, hearty woman, in labor with her third child. She is always severely and tediously sick. I thought it a good case for the use of chloroform, but did not succeed in bringing her into a state of anaesthesia, nor would she acknowledge she derived the least relief from it. I thought I made a fair trial, having given a quantity that I know, by frequent trials, would have put me soundly asleep. The second case was one who had been sick for some weeks, and who tried her best to breath it; but with all the instructions I could give her, she did not learn to inhale it freely. Still, two or three times I thought she got enough to have some effect; but as she said not, I declined giving more. In the third case I had an opportunity to make only a moderately good, but unsuccessful trial, when the birth of the child prevented further attempts.

*Of the case in which chloroform was given for* 14¾ *hours.*—It was that of a delicate woman with her first child. She had been nearly 2½ days in constant pain wnen its inhalation was commenced. She did not require much at a time to ease her pains. Three ounces were consumed, which, when we consider the length of time it was being given shows the smallness of the dose used. The labor was finally terminated with the forceps. The relief obtained from the chloroform during that tedious day may be judged of by her remark when I announced to her the neccesity for the use of the instruments: "well, doctor, just as you are of a mind to, if you will give me plenty of chloroform." It is hardly necessary to add that it was given according to her wishes, and the child taken away without there being, on her part, the least sensibility to the operation.

*Of the seventeen Forcep cases.*—Five were rendered totally insensible at the time of the operation. The delivery was rapid, requiring in each but two or three efforts at traction. In four cases chloroform was given during the operation, but not to full anaesthesia. In eight cases chloroform had been given some time during the progress of the labor. Four were cases of complication and danger, and it was discontinued that any unpleasant results which might happen should not be refered to it. In the remaining four cases the delivery was slow, and required my whole attention without the distraction which would result from watching also the use of cloroform.

Of the seventeen cases, fourteen were first labors.

*Of the case of Craniotomy.*—This was necessary on account of mal-position and impaction of the head within the pelvis. The woman, during a portion of the labor only, had chloroform, but with only partial relief, as it was not freely administered. None was given during the operation.

*Of the case of Turning.*—The following case of turning is the only one in which I have given chloroform. The woman was a patient of Dr. Dayton, of Black Rock. He was hastily summoned to her in the morning of August 11th, 1849. He found that the waters had escaped while she was on the close stool, and the child's right hand was protruding into the world. The patient finding that something was wrong, and thinking herself certain to die, notwithstanding the Doctor's assurance of her entire safety, gave herself up to the most immoderate weeping, and other evidences of excessive grief. Dr. Dayton attempted to turn the child and deliver, but such was her restlessness and violent outcries, that he desisted and sent for me. It was four hours after the coming down of the hand before I reached the house. The woman had in a great measure got ove her alarm, and was waiting very patiently. There had been no return of pain during all this time. The prolapsed member was much swollen; a spasmodic contraction of fingers on my taking hold of the hand, proved the child to be living.

After having her properly placed, Dr. Dayton administered the chloroform slowly, but in a full dose, so she soon became entirely anaesthetized, when the sponge was removed. Not over twelve or fifteen minutes were occupied in turning and delivering the child. It was accomplished with great ease, although the limbs were so folded upon themselves that I had to reach into the very fundus of the womb before I could conveniently seize one limb. The child turned readily, and the arm gradually receded, but not entirely, until I had withdrawn the left foot (so the right hand and left foot were at the same instant without the vulva), and soon afterwards the child was delivered living. The patient laid in the most quiet manner all this time; she did not utter a single cry or moan. Once she became for an instant a little restless, but three or four additional inspirations of the chloroform perfectly quieted her. There was no perceivable contraction of the voluntary muscles during the delivery. The uterus contracted down well, and the after-birth was delivered in due time, and without hemorrhage. The patient did not wake from her sleep for some four or five minutes after the child was born. She recovered without a bad symptom.

It has been my lot a number of times to deliver by turning, but never before have I accomplished it, at the full time, with one-half the ease and rapidity I was enabled to do in this case.

*First labors*—88 *cases.*—The large proportion of cases of first labor relative to the whole number of cases of labor, as they occur in ordinary practice, conclusively shows that in the choice of cases for the use of the remedy, I have been governed solely by their tediousness and severity. In but a few instances have I yielded to the wishes of my patients, and given it to those who were not suffering with considerable severity. In fact, except in refusing to give it in some cases where I apprehended sickness, I have never made any selection as such, of cases in which to administer it.

*Still-born children*—14 *cases.*—*One* was after delivery by craniotomy. *Two* were delivered putrid. One was an extraordinarily large child, which presented the breach where delivery was very slow, especially in the extraction of the head. Chloroform was not given at the time of delivery, nor had it been for an hour or more before. 1. *Two* were twins, at four or four and a half months. *One* died from prolapsus of the cord. *Two* were hydrocephalic children, and both with spina bifida. *One* was a child delivered by the forceps, and which had undoubtedly been dead for some hours. *One* died during a labor which was uncommonly severe and prolonged, from a slow dilation of the vagina and rigidity of the perineum. Chloroform was given for some nine hours. It was a first labor and the child was large. *Two* of the children were two or three months premature. In the second of these, death was undoubtedly caused by the separation of the placenta, which was the first and exciting cause of the labor itself. In the other, no cause can be given why the child was perfectly still-born, it being only two months premature. The *fourteenth* was a child also perfectly still-born, but in which no cause could be assigned.

Besides these, two children lived 24 and 36 hours, respectively, after birth. The first was an acephalous child (and curiously enough, this want of a brain has actually been laid to the chloroform), and the other was a case of severe labor, in which delivery was accomplished by the forceps. The child was badly asphyxiated, but after an hour's assiduous attention, got to breathing tolerably well; but the next day it took spasms and died. Its pulse was never very perceptible at its wrist.

In judging of the effect of chloroform upon the child, the case of its use for 14¾ hours ought to be borne in mind, as well as a case in which it was given over five hours, and the patient's system became so saturated with it, that I detected it in her breath one hour after the conclusion of the labor. In both of these cases, the children cried out lustily the instant of birth.

*Flooding after the use of chloroform—seven cases.*—The cases of flooding admit of being divided into two classes; first, those in which it occurred before the delivery of the placenta, from its partial separation; and second, those which were caused by want of a firm, tonic contraction of the uterus. Three of the seven cases come under the first head, and four under the second. Of the first class, *one*, after a natural and easy labor, flooding frightfully. The flowing came on immediately on the birth of the child. Scarcely waiting to tie the cord, I introduced my hand, and delivered the placenta. The whole time from the delivery of the child to that of the placenta was scarcely three minutes; but the flooding was so severe, and the faintness so prolonged, that for an hour afterwards I was seriously alarmed lest I should lose my patient. She had had a similar flooding on the birth of the child before this, but had not informed me—this being the first time I had attended her. She got along very well for a few days, when she was attacked with phlegmasia dolens, which also she had had after the previous confinement, but when she did not have chloroform. The only reason chloroform was given this time was because she wanted it, and I saw no objections to her having it.

The second case was very similar to the first, except the flooding was not so severe, nor the faintness so great, nor was there any phlegmasia dolens.

The third case was one where the patient had been confined to her room by sickness for some days. Her labor proving tedious, and seemingly to her unbearable, notwithstanding her sickly condition, I gave her chloroform for two hours before the birth of the child. The flooding required the manual delivery of the placenta, after which, for an hour, there was quite a free discharge. Her own health was such that she was not allowed to nurse her child, but after a rather tedious convalescence she quite recovered her health.

Of the four cases belonging to the second class, the first was very severe from the difficulty of securing the contraction of the uterus, and gave me considerable anxiety for a while. The labor had been severe, and she had taken pretty freely of chloroform. She was one who always flooded badly after labor, but which at the time, I did not know. In none of the three other cases was it severe enough to create anxiety, except in so far as we dislike to see such losses of blood from its likelihood to weaken our patients.

In August, 1849, I gave chloroform to a patient also subject to hemorrhage. It was her third confinement. She had flooded badly after each of the previous ones. I administered pretty freely of an infusion of ergot in the intervals of the pains, besides giving the chloroform, and succeeded both in preventing the flooding and in easing her pains.

*Hysteritis after the use of chloroform—six cases.*—In one case peritonitis supervened upon hysteritis, and the patient died six and a half days after labor. She was an exceedingly frail woman, and had suffered daily for six months before labor. Three or four times during this period, she had such severe and continued pain that she was thought certainly to be about to miscarry. Her labor was very sharp and painful. Chloroform was given with great relief for three hours before its termination. Once having experienced the relief it afforded, she would not do without it. She appeared very well the next day, except in having too great a warmth of surface and a pulse at one hundred. The next day after this she got severe pains through the pelvis, which rapidly extended over the bowels with all the signs of peritonitis. No efforts accomplished more than partially to relieve her. This is the only case within my experience which has proved fatal after taking chloroform.

One case had puerperal fever, but apparently uncom plicated with inflammation. The left groin was the only seat of any local pain. She was fully depleted and gradually recovered. She took chloroform for half an hour.

One case had hysteritis only moderately, and it was easily controlled by remedies. She took chloroform freely for two hours.

Three had hysterits severely, and were all at some period of their sickness, in great danger. The one most severely sick took chloroform not over fifteen minutes, just at the close of the labor. Another was after the operation of craniotomy; chloroform had not been freely given. The last one of the three took chloroform for some hours, and was finally delivered with the forceps.

A case of phlegmasia dolens is referred to under the head of cases of flooding.

These are the only cases I have seen of inflammation of any of the internal organs of the body after the use of chloroform.

*Of bad health after the use of chloroform*—25 *cases.*—It has often been said that women do not have a good getting up after the use of chloroform. I have carefully scanned my list, for all, as far as I know, who have not done perfectly well after its use. I make out a list of 25 cases. These I classify as follows:

1st. Those who not only had been sick previous to confinement, but were sick at the time, 9 cases.

2d. Those who suffered afterwards from a great degree of constipation, 2 cases.)

3d. Those whose convalesence was protracted after flooding, 2 cases.

4th. Those who always became feeble when nursing, 1 case.

5th. Those who suffered from prolapsus uteri, 1 case.

6th. Those who had had long and severe labors and recovered slowly the first four or five weeks, 4 cases. (Three of these were forceps cases.

7th. Those who were kept sick and feeble for three or four weeks from abscess of the breast, 2 cases.

8th. Those who were feeble after having had hysteritis, 4 cases.

Of these twenty-five cases, one died three years ago of epidemic dysentery (three months after having taken chloroform), and one died last summer of cholera (nine months after taking it). These are the only deaths in this list. Of the remainder, one is now suffering from a scrofulous abscess of the neck; it first showed itself six months after her confinement. Two have had some cough, and both being of feeble constitutions, give me some anxiety about their future health. One has moved to the west, and I believe her to be well. The other 19 cases I know all to be in good health.

My own opinion is that in not one of these instances ought the ill health to be charged to the use of the chloroform.

I have seen many instructive illustrations of this disposition to refer any unpleasant symptom to the use of chloroform, of which two or three may as well be detailed here: A woman whom I was attending last summer wanted chloroform, but on account of her husband being fearful of its safety, it was not given. Two or three months afterwards she was complaining to me of her eyesight being dim, and said it had been so ever since her sickness. Had she taken chloroform, both herself and friends would, in all probability, have attributed it to that, and not the least weight would have been given to any doubts or denials I might have expressed or made.—Another, who was only prevented from taking it by my absence, had, two hours after the labor, a dizziness and entire loss of sight for a few minutes, which quite alarmed her. She confessed she would have attributed it all to chloroform had she taken it. I was lately consulted by a patient who is threatened with a rapid decline, and who refers the commencement of her ill health to the time of her first confinement. She has never since been as well as she was before. I was thankful she had not taken chloroform. The truth is, that like the mercurials and the sulphate of quinia, it will have to bear, both now and hereafter, a deal of obloquy and evil report wholly unmerited.

